# Sterile Acellular Dermal Collagen as a Treatment for Rippling Deformity of Breast

**DOI:** 10.1155/2014/876254

**Published:** 2014-12-25

**Authors:** Brittany Busse, Hakan Orbay, David E. Sahar

**Affiliations:** ^1^Department of Surgery, UC Davis School of Medicine, Sacramento, CA 95817, USA; ^2^Division of Plastic Surgery, UC Davis School of Medicine, Sacramento, CA 95817, USA; ^3^Division of Plastic Surgery, University of California Davis Medical Center, Cypress Building 2221 Stockton Boulevard, Suite 2125, Sacramento, CA 95817, USA

## Abstract

Prosthetic implants are frequently used for breast augmentation and breast reconstruction following mastectomy. Unfortunately, long-term aesthetic results of prosthetic breast restoration may be hindered by complications such as rippling, capsular contracture, and implant malposition. The advent of use of acellular dermal matrices has greatly improved the outcomes of prosthetic breast reconstruction. We describe a case of rippling deformity of breast that was treated using an acellular dermal matrix product, AlloMax. The patient presented with visible rippling of bilateral prosthetic breast implants as well as significant asymmetry of the breasts after multiple excisional biopsies for right breast ductal carcinoma in situ. A 6 × 10 cm piece of AlloMax was placed on the medial aspect of each breast between the implant and the skin flap. Follow-up was performed at 1 week, 3 months, and 1 year following the procedure. The patient recovered well from the surgery and there were no complications. At her first postoperative follow-up the patient was extremely satisfied with the result. At her 3-month and 1-year follow-up she had no recurrence of her previous deformity and no new deformity.

## 1. Introduction

Prosthetic implant use for cosmetic breast augmentation and postmastectomy breast reconstruction is a common procedure. Unfortunately, there are several long-term complications of breast restoration with prosthetic implants, such as rippling, capsular contracture, implant malposition, bottoming out, implant exposure, and symmastia. A majority of these complications are due to thinning of the overlying skin as a result of expansion process, periprosthetic atrophy, formation of a thin or atrophic capsule, inadequate filling or incorrect placement of the implant, or overgenerous formation of the breast pocket [1, 2]. Rippling is the appearance of vertical folds on the skin of a patient with a prosthetic breast implant and is most visible when the patient is in an upright and/or forward-leaning position. It is noted in about 0–10% of patients after prosthetic breast implant augmentation/reconstruction [3–5] and 5–50% of cases require reoperation [4].

Rippling and other aesthetic complications related with breast implants have been very difficult to manage with revisionary surgery. High recurrence rates and low patient satisfaction are frequent. However, reconstructive surgeons achieved greatly improved outcomes since the advent of use of acellular dermal matrix (ADM) [3, 4]. ADM adds an extra layer of tissue between the implant and the skin and therefore decreases the visibility of implant. A review of the current literature includes many references to the use of AlloDerm (Life-Cell Corporation, Branchburg, NJ), the first commercially available ADM product, to repair breast rippling associated with prosthetic implants [5–7] but there is currently no literature on the use of AlloMax (Bard-Davol, Warwick, RI) for the same indication. AlloMax is a similar ADM product; however, in contrast to AlloDerm, AlloMax is terminally sterilized using Tutoplast process, which is a proprietary process for decellularization, sterilization, and viral inactivation.

Herein, we describe a case in which we have treated the bilateral breast rippling deformity of a patient using AlloMax.

## 2. Case Report

**Figure 1:**
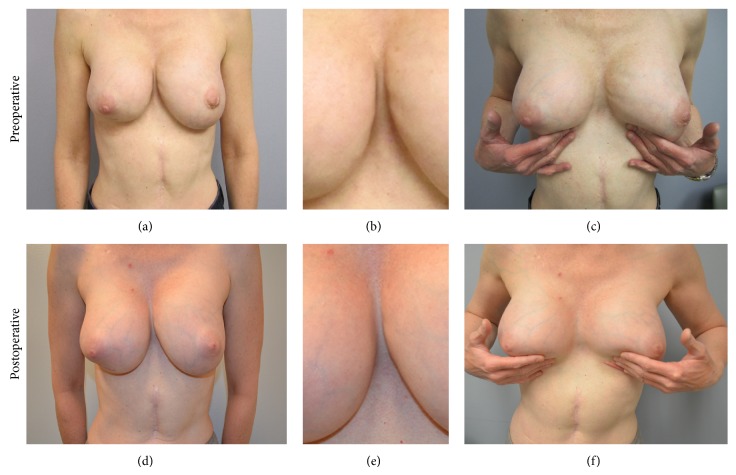
Preoperatively IMFs of the patient were asymmetric (a) and rippling was noticeable in the medial aspects of her breasts ((b) and (c)). Postoperatively IMFs were at the same level (d) and rippling deformity disappeared ((e) and (f)).

The patient was a 47-year-old woman who presented with visible rippling of bilateral prosthetic breast implants after multiple excisional biopsies for right breast ductal carcinoma in situ (DCIS). She had placement of bilateral saline prosthetic implants for cosmetic breast augmentation from a negative A to a C in 2000 with good results and no immediate postoperative complications. She was diagnosed with DCIS of the right breast in 2012 and underwent wire-guided excisional biopsy of the right inferolateral quadrant with subsequent reexcision for positive margins. Following these procedures, she noted significant breast asymmetry and rippling deformity in both of her breasts. On admission, she had asymmetric breasts with a well-healed horizontal scar on her right breast 1-2 cm above the inframammary fold (IMF). The IMF to nipple distance was significantly decreased on the right side compared to the left side and right nipple was more ptotic (Figure 1(a)). Rippling was visible along the medial borders of the breasts bilaterally (Figures 1(b) and 1(c)).

**Figure 2:**
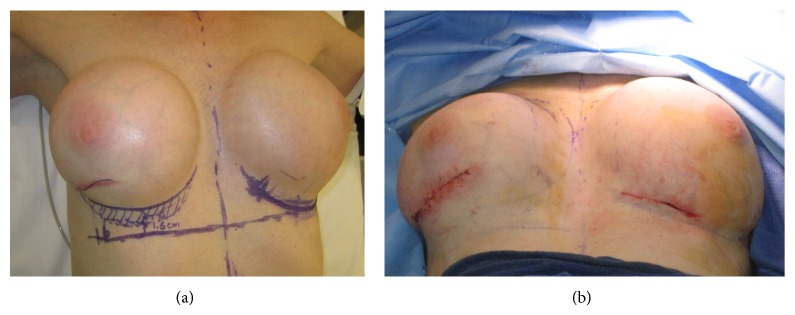
The picture shows the level of discrepancy between the IMFs on the left and right breasts (a) before the operation and the immediate postoperative results following the wound closure.

Because of the patient's thin skin we decided to proceed with right breast augmentation with the exchange of implant to a larger size implant (275 to 350 mL) and placement of AlloMax bilaterally to improve the rippling. In the operating room, attention was first turned to the right breast, where a hypertrophic scar from previous biopsy site was noted (Figure 2(a)). We made an incision to excise the hypertrophic scar, and incision was carried down to the subcutaneous fascia through the capsule. We opened the capsule and removed the existing 275 cc normal saline implant (Mentor, Santa Barbara, CA). We also performed a medial and superior capsulectomy. For reconstruction, we soaked a 6 × 20 cm AlloMax dermal matrix sheath in normal saline for 5 minutes. The thickness of AlloMax was between 0.8 and 1.8 mm. We cut it into half and placed a 6 × 10 cm segment on the medial aspect of the left breast between the implant and the skin flap. The distal aspect remained free to prevent tethering. We replaced the original implant with a smooth, round, and moderate profile and 300 cc saline breast implant with a diaphragm valve (Mentor, Santa Barbara, CA). We expanded the implant to a 350 cc total volume, approximately 75 cc above the original implant, and closed the incision in layers. Attention was then turned to the left breast where we opened the capsule and removed the existing implant intact. We performed a capsulectomy on medial aspect and placed the other half of AlloMax, measuring approximately 6 × 10 cm, between the implant and the skin flap. We secured the AlloMax proximally to the chest wall using 4-0 absorbable suture in interrupted fashion. After irrigation, we placed the implant back into the pocket and closed the wound in layers (Figure 2(b)). The patient was discharged from the postanesthesia care unit the same day with no apparent complications.

The patient returned for a postoperative follow-up visit 5 days following her procedure. There were no wound related complications. At 3-month follow-up the incisions were well-healed with no hypertrophic scaring. There was no recurrence of rippling and IMFs were symmetric bilaterally. At 1-year follow-up patient's breasts were symmetric bilaterally with no visible rippling of the medial or inferolateral edge of either breast (Figures 1(d), 1(e), and 1(f)). The patient was satisfied with the overall result.

## 3. Discussion

The use of ADM as a filler for correction of implant-related breast deformity has been commonplace for many years [3, 6]. Revisionary procedures performed using ADM improved the appearance of rippling in 85% of the patients [6] with failure rates as low as 5% [9]. ADM is placed by either capsular onlay or lower pole suspension technique to revise rippling deformity. In the dermal onlay technique, the implant is removed and placed in antibiotic solution. The ADM is then sutured into place between the capsule and the implant, same as the technique we have used in this case. The lower pole suspension technique is very similar to placement of an ADM sling in primary procedures: the ADM is sutured to the IMF and the inferior border of the pectoralis major muscle to support the inferior aspect of the implant. This elevates the prosthetic implant and ameliorates underfilling of the upper pole.

The most extensively studied ADM is AlloDerm, a human cadaveric split thickness dermis manufactured by Life-Cell Corporation. AlloDerm is referenced in 572 articles in the PubMed Database, 77 of which describe its use in breast reconstruction. AlloMax, an acellular human dermal graft that is described by the manufacturer as “acellular dermal collagen,” is similar to AlloDerm. However AlloMax is prepared using a unique process named Tutoplast process, first invented by Tutogen (later merged with RTI Biologics Inc., Alachua, FL), which is a proprietary process for decellularization, sterilization, and viral inactivation of the graft material [10, 11]. The Tutoplast process is a five-step procedure that results in terminal sterilization of the product, and currently AlloMax is one of the two terminally sterilized ADMs that are commercially available [12].

There are other subtle differences between AlloDerm and AlloMax as noted by the manufacturer. Some of the key benefits of AlloMax as compared to AlloDerm are the shorter preparation time, terminal sterility, and the fact that no orientation is necessary for implantation [13] (Table 1). Also because the Tutoplast process negates the need for antibiotic soaking prior to implantation there are no allergenic contraindications to implantation at this time. Although the cost per cm^2^ for AlloMax is slightly higher than for AlloDerm, there are certain cost saving measures built into its use, such as the reduction in preparation time, single hydration step, and longer shelf life. Additionally, AlloDerm was associated with increased incidence of postoperative wound infections after breast reconstruction, and it is the main etiologic factor behind a sterile, chronic cellulitis known as “red breast syndrome” [8, 14, 15]. The syndrome resolves without treatment but results in clinical confusion and patient discomfort. There are no reports relating the occurrence of “red breast syndrome” with AlloMax. Despite all the advantages, it is still controversial if the integration of AlloMax to the recipient bed is impeded by the Tutoplast processes [12].

A terminally sterilized form of AlloDerm (RTU (ready to use) AlloDerm) has been introduced and utilized recently to address the issues related with the use of aseptic (nonsterile) AlloDerm [16, 17]. Overall infection rates in patients undergoing reconstruction with RTU AlloDerm decreased significantly. However, as in case of AlloMax, the effect of the terminal sterilization procedure on the incorporation of the RTU AlloDerm to the recipient tissues remains controversial [16].

The other ADM products that can be used in breast reconstruction are Strattice, DermaMatrix, SurgiMend, Veritas, and FlexHD. Among these only the DermaMatrix and Flex HD are human origin ADMs. Strattice, SurgiMend, and Veritas are obtained from various animal resources which can be considered as a drawback in their clinical use. Strattice and FlexHD are mostly used for breast reconstruction while the other products are generally used for the reconstruction of other deformities, such as abdominal hernia. Strattice is obtained from porcine skin which is less desirable than human skin but on the other hand it is thicker and stronger than AlloDerm and it is a terminally sterile product that is available in larger pieces (up to 20 × 25 cm), potentially minimizing wound dehiscence. FlexHD is described as acellular hydrated dermis and is derived from cadaveric human allograft skin. It is delivered prehydrated and does not require refrigeration; furthermore, it exhibits resistance to stretch and biomechanical strength resulting in a tissue graft that is ready for immediate use, potentially reducing operation room time [18].

Another alternative for the treatment for the rippling deformity of breast that should be discussed is fat grafting. Ease of fat harvest and abundance of donor sites in addition to the discovery of vasculogenic stem cells within adipose tissue increased the popularity of fat grafting in the last decade. The major drawbacks of the fat grafting, however, are cyst formation and benign calcifications in the breast that may obscure the diagnosis of future malignant lesions. Therefore, the patients should be selected carefully and if possible fat grafting should be avoided in patients with history or increased risk of breast cancer such as the patient in this case report.

The correct use and placement of an ADM are imperative when using the material for breast reconstruction or other indications. The learning curve can be quite high and includes attention to such aspects as proper rehydration of the material, proper fit, and elimination of dead space around and within the material [4]. AlloMax has specific use instructions that may differ in some respects from other ADMs so it is important to get familiarized with the material before proceeding with clinical application.

## 4. Conclusion

This single patient case report demonstrates good aesthetic outcome up to 1 year after the operation with the use of AlloMax to correct breast rippling deformity. Our group is currently enrolling patients for a randomized controlled trial to compare the infection rates between the breast reconstruction cases performed by using AlloMax or AlloDerm. We will also observe the tissue incorporation of the two materials in order to offer a solution to the ongoing controversy on this topic.

## Figures and Tables

**Table 1:** The key differences between AlloDerm and AlloMax (adapted from [8]).

Product	Source	Prep. time	Preparation	Refrigeration	Sterility	Orientation	Cross-linking	Shelf life	Cost/cm^2^
AlloDerm	Human	10–40 min	2 baths of warm NS^1^ or LR^2^; the second should include antibiotics	No	No	Yes	No	2 y	$28
AlloMax	Human	3 min	Room temp. NS	No	Yes	No	No	5 y	$32.38

^1^NS: normal saline; ^2^LR: Lactated Ringer's.
